# Impact of negative emotions on upper gastrointestinal diseases: A Mendel randomization study

**DOI:** 10.1371/journal.pone.0304121

**Published:** 2024-07-12

**Authors:** Nan Gao, Zhun Yu, Yu Fan, Xue Jiang, Ting Hu

**Affiliations:** Changchun University of Chinese Medicine, Changchun, China; University of Texas School of Public Health, UNITED STATES

## Abstract

Mendelian randomization method is a powerful tool in epidemiological research. The core idea is to use genetic variation as a tool to assess the causal relationship between risk factors and specific diseases. Confounding factors are important interference factors for causal inference in epidemiological studies, and genetic variation in Mendelian randomization studies follows the principle of random distribution of alleles to offspring, which is similar to randomized controlled trials. Mendel ’s randomization method can effectively avoid the confounding factors, reverse causality in observational studies and the representativeness and feasibility of randomized controlled trials. Previous observational studies have reported a relationship between negative emotions and upper gastrointestinal disease. However, whether this relationship is causal remains unclear. We aimed to evaluate the causal relationship between negative emotions and upper gastrointestinal diseases using two-sample Mendelian randomization (MR). Three sets of genetic instruments from the database were obtained for analysis, including 12 anxiety-related single nucleotide polymorphisms (SNPs), 46 depression-related SNPs, and 58 nervous-related SNPs. SNPs were filtered using the Phenoscanner website, and the inverse variance weighted method, weighted median method, MR-Egger regression, MR pleiotropy residual sum, and outlier test were used for analysis. In inverse variance weighted analysis, anxiety and depression had an effect on gastroduodenal ulcer (p = 2.849×10^−3^, β = 4.908, 95% CI = 1.684–8.132; and p = 6.457×10^−4^, β = 1.767, 95% CI = 0.752–2.782, respectively). Additionally, depression had an effect on diseases of the esophagus, stomach, and duodenum (p = 3.498×10^−5^, β = 0.926, 95% CI = 0.487–1.364). Cochran’s Q-derived p-values were 0.457, 0.603, and 0.643, and MR-Egger intercept-derived p-values were 0.697, 0.294, and 0.362, respectively. Here, we show that anxiety and depression have a causal relationship with gastroduodenal ulcers, and depression has a causal relationship with diseases of the esophagus, stomach, and duodenum.

## Introduction

Gastrointestinal disease is common, with a previous study demonstrating that approximately half of all young people experienced one or more types of gastrointestinal symptoms in the week prior to survey [1]. These gastrointestinal symptoms are mostly related to high levels of perceived stress and reactivity, negative coping, and anxiety sensitivity [2]. Another study showed that the incidence rates of irritable bowel syndrome and dyspepsia are increased in patients suffering from anxiety, depression, and other negative emotions [3]. The gut-brain axis provides a theoretical basis for revealing the relationship between psychological pressure, cognitive emotional processes, and gastrointestinal symptoms [4]. The upper and lower digestive tracts have different effects on the brain and gut because of their different roles in human physiological activities. The consequences of different negative emotions also vary.

Previous study has examined the relationship between anxiety and functional dyspepsia, with many demonstrating that anxiety is related to functional dyspepsia and postprandial pain syndrome but not epigastric pain syndrome [5]. This study found that depression had little effect on functional dyspepsia. A meta-analysis suggested that oxidative stress plays a role in depression [6]. Therefore, when depression occurs, excessive reactive oxygen species (ROS) production impairs the body’s clearance ability, which may lead to the occurrence of primary gastric cancer [7]. Some studies have found that depression can increase the invasion and metastasis of gastric cancer [8], thereby showing a correlation between negative emotions and upper digestive tract diseases. However, owing to problems related to confounding factors and reverse causality in observational studies, it is important to determine the causal relationship between negative emotions and upper digestive tract diseases.

Mendelian randomization (MR) is a genetic epidemiological design that can effectively avoid the occurrence of confounding factors and reverse causality [9]. To the best of our knowledge, two-sample MR has not been used to evaluate the causal relationship between negative emotions and upper gastrointestinal disease. We used aggregated statistical data from a large-scale genome-wide association study (GWAS) of annealing, depression, and nervousness to reveal the causal impact of negative emotions on the risk of upper gastrointestinal diseases.

## Method

In this study, as we reanalyzed data obtained from previous research results; therefore, no additional ethical approval was required.

### GWAS summary data for negative emotions

**Fig 1:**
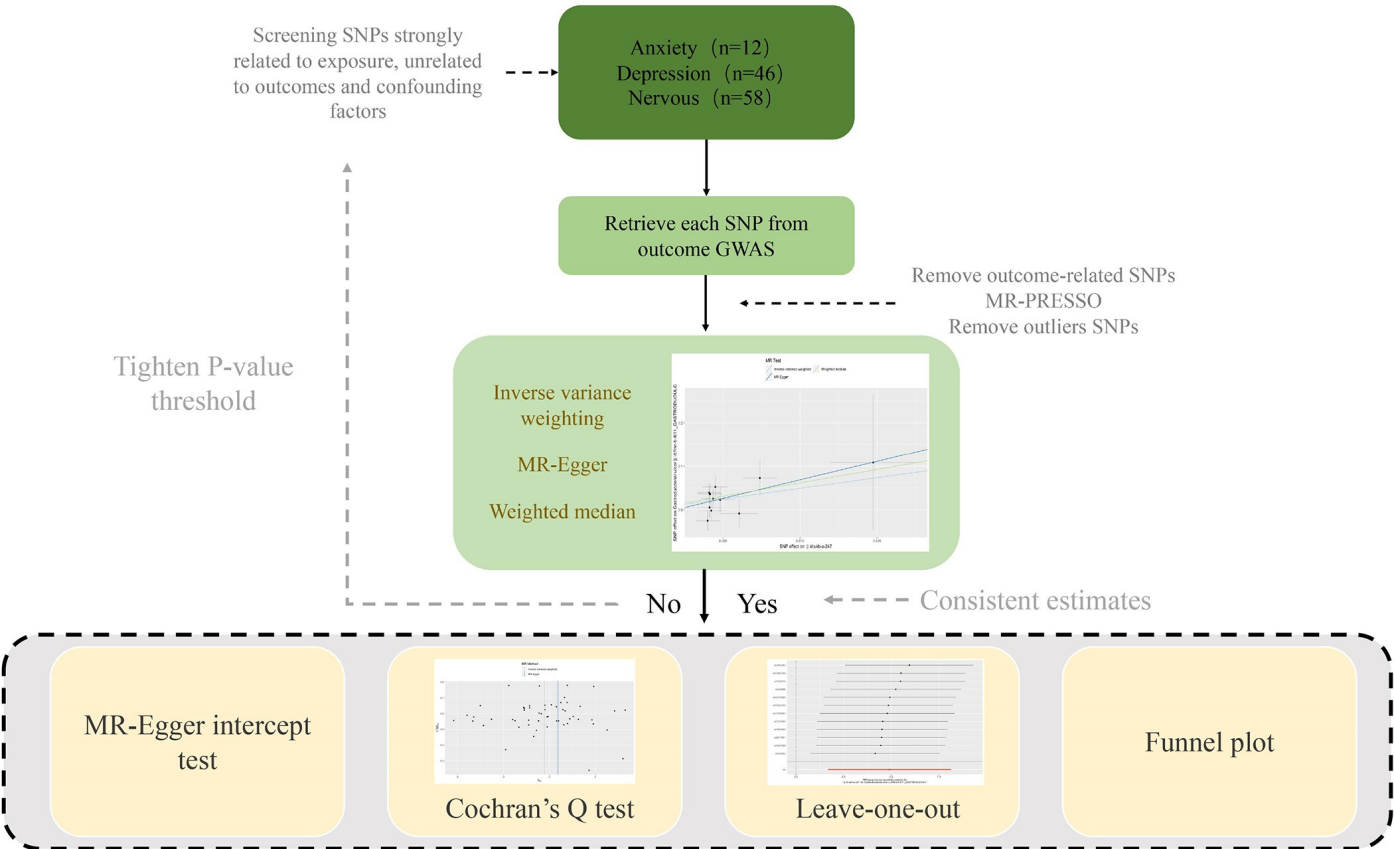
Workflow of Mendelian randomization study.

An overview of the study design is shown in Fig 1. We obtained genetic instruments for negative emotions, such as anxiety, depression, and nervousness, which can be used as exposure for subsequent research. The summary data of a recent GWAS comes from the IEU OpenGWAS project (https://gwas.mrcieu.ac.uk/). Among the data, those relating to anxiety comprised 53414 cases and 407288 control samples from the European population. The summary-level GWAS data correlated with depression were obtained from a GWAS of depression phenotypes in the UK Biobank consisting of 501726 individuals, among which, 113769 were included in the current research [10]. The summary-level GWAS data correlated with nervousness were obtained from a UK biobank study containing 503325 participants [11]. The relevant content is summarized in S1 Table. The proportion of variance in negative-induced emotion explained by each single nucleotide polymorphism (SNP) was estimated using the R^2^ value [12], and the instrumental strength of each SNP was assessed using the F-statistic [13]. The variability and F-values of these SNPs are listed in S2 Table. The F-values of all SNPs were greater than 10.

It is revealing causality from negative emotions on upper gastrointestinal diseases. IVW, inverse variance weighted; MR, Mendelian randomization; MR-PRESSO, MR Pleiotropy RESidual Sum and Outlier; SNP, single-nucleotide polymorphisms.

### GWAS summary data for upper digestive tract diseases

The GWAS related to upper gastrointestinal diseases used in this study originated from the IEU OpenGWAS project (https://gwas.mrcieu.ac.uk/) (S1 Table).

### Selection of tool variables

We adopted a unified selection standard for genetic variation in anxiety, depression, and nervousness. To obtain as many SNPs related to negative emotions as possible, we set p < 5 × 10^−7^ as the threshold for screening [14], and SNPs meeting linkage disequilibrium [LD] r2 < 0.01 were used as independent genetic variations. To avoid possible SNPs related to upper gastrointestinal diseases, we rechecked the SNPs using the Phenoscan website (http://www.phenoscanner.medschl.cam.ac.uk/).

### Statistical analysis

After harmonization of the effect alleles across the GWASs of negative-induced emotion and upper digestive tract diseases, we used several MR approaches to determine MR estimates of negative-induced emotion for upper digestive tract diseases, namely the inverse variance weight (IVW), weighted median, and MR-Egger. In this study, random effects inverse-variance weighted (IVW), weighted median and Mr-Egger methods were used to perform two-sample Mendelian randomization analysis, and the OR value was used to evaluate the potential causal relationship between negative emotions and upper gastrointestinal diseases. The traditional inverse variance-weighted analysis method may be affected by invalid instrument bias or pleiotropic effects. Therefore, this study passed the sensitivity test. Perceptual analysis was used to test the validity and robustness of IVW results.

IVW has a strong ability to detect causality, but specifically requires genetic variation to affect the target outcome only through exposure. Therefore, it is possible to produce pleiotropy and cause a bias in the estimation of the effect value. As such, we also needed two other methods to test the reliability and stability of the results. Owing to the differences in the analysis platform and experimental conditions, MR may have heterogeneity. Cochran’s Q test was used to identify heterogeneity, and the test result was considered to have heterogeneity if p < 0.05. We used MR-Egger regression analysis to evaluate the bias of gene pleiotropy. The smaller the regression intercept, the smaller the pleiotropy. This study was implemented using the software packages TwoSampleMR (version 0.4.25) and MRPRESSO (version 1.0) in R (version 4.1.2).

### Results

Through screening, 12 SNPs were found to be correlated with anxiety, 46 with depression, and 58 with nervousness. Details of these SNPs, including the variability and F-values, are provided in S2 Table. The F-values of all SNPs were greater than 10, indicating that these instruments have a strong correlation with upper gastrointestinal diseases.

**Fig 2:**
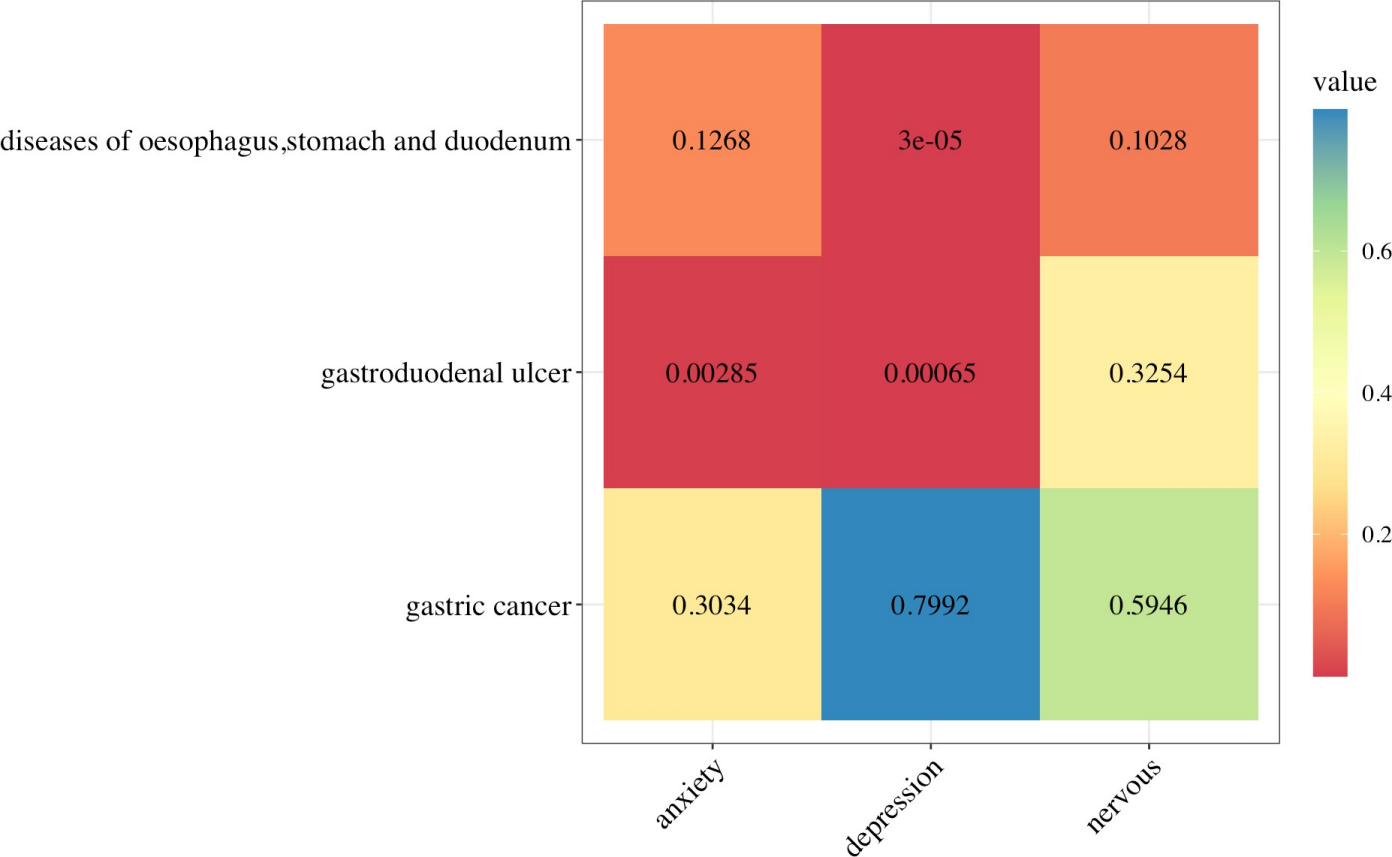
Inverse variance weighted (IVW) estimates from anxiety, depression, and nervousness on upper gastrointestinal diseases. The color of each block represents the IVW-derived p-values of each MR analysis. P-values < 0.05 are shown in red and those > 0.05 are shown in yellow, green, or blue.

**Fig 3:**
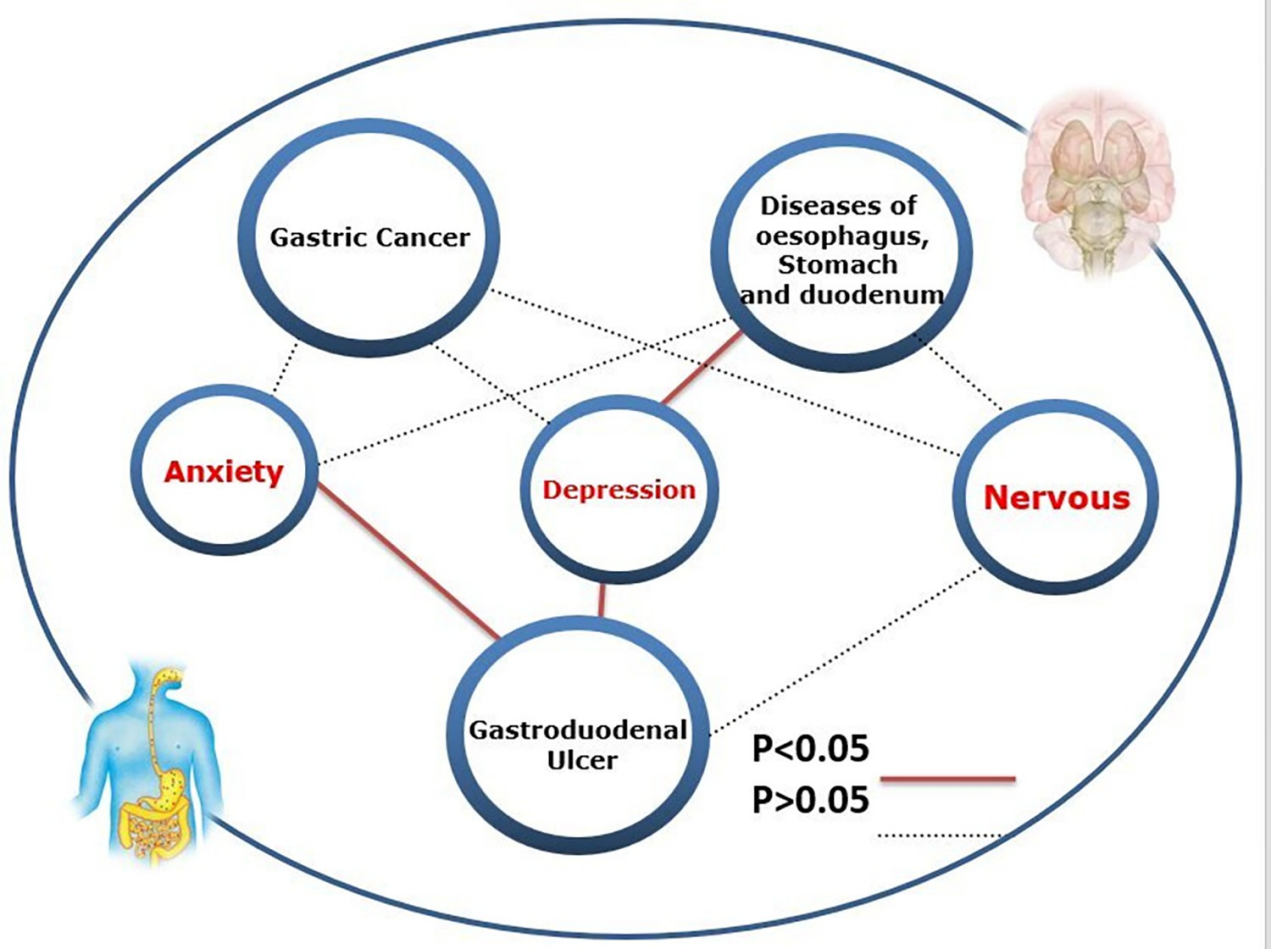
Gut–brain–axis. Using a two-sample Mendelian randomization framework, we reveal that negative emotions causally influence upper gastrointestinal diseases, supporting the existence of the gut–brain–axis. P-value of the IVW estimate < 0.05 is set as significant, represented as a solid line whereas > 0.05 is set as nominal significant, represented in dotted line.).

We used MR to study the influence of three negative emotions (anxiety, depression, and nervousness) on gastric cancer; gastroduodenal ulcer; and esophageal, gastric, and duodenal diseases It shows in Fig 2. Fig 3 presents the upper gastrointestinal diseases affected by negative emotions (., which can affect the occurrence of gastroduodenal ulcers (β = 4.908, 95% confidence interval [CI]:1.684–8.132, p = 2.849×10^−3^), while depression can affect the occurrence of gastroduodenal ulcer (β = 1.767, 95% CI 0.752–2.782, p = 6.457×10^−4^) and diseases of the esophagus, stomach, and duodenum (β = 0.926, 95% CI: 0.487–1.364, p = 3.498×10^−5^) (Table 1). The p-value obtained from Cochran’s Q was greater than 0.05, indicating that there was no heterogeneity, and the p-value of the MR Egger intercept was greater than 0.05. No abnormal values were found in the MR-PRESSO, leave-one-out plot, and fund plot (S1–S3 Figs).

**Table 1:** Significant Mendelian randomization estimates from negative emotions on upper gastrointestinal diseases.

Exposure	Outcome	IVW-derived p-value	*Β* (95% Confidence interval)	Cochran’s Q-derived p-value	MR-Egger intercept-derived p-value
Anxiety	Gastroduodenal ulcer	2.849×10^−3^	4.908 (1.684–8.132)	0.457	0.697
Depression	Gastroduodenal ulcer	6.457×10^−4^	1.767 (0.752–2.782)	0.603	0.294
	diseases of esophagus, stomach and duodenum	3.498×10^−5^	0.926 (0.487–1.364)	0.643	0.362

We screened the SNPs using Phenoscanner to test whether the risk factors violated the significance assessment. As a result, we removed 51 SNPs related to upper gastrointestinal diseases, including abdominal pain, alcohol consumption, smoking, BMI, and oral aspirin (S3 Table). The results before and after removing these SNPs were consistent; the causal relationship between the apparent negative emotions and upper gastrointestinal diseases was not affected by potential risk factors.

## Discussion

We conducted an MR analysis on the causal relationship between anxiety, depression, and nervousness and upper gastrointestinal diseases. To this end, we systematically evaluated the relationship between genetic prediction of negative emotions and upper gastrointestinal diseases. The results showed that anxiety and depression can affect gastroduodenal ulcers and that depression can affect esophageal, gastric, and duodenal diseases. These results further confirm the existence of the gut-brain axis, providing a basis for the influence of emotion on upper gastrointestinal diseases.

According to previous studies, people with negative emotions, such as anxiety, depression, and nervousness, are at higher risk of gastrointestinal diseases, such as digestive dysfunction [15, 16]. However, the causal relationship between negative emotions and upper gastrointestinal disease has not yet been fully clarified. The upper digestive tract refers to the part above the flexor ligament, mainly the esophagus, stomach, and duodenum. We selected gastric cancer and gastroduodenal ulcer with a high incidence rate for a single disease evaluation, and also studied the diseases of the esophagus, stomach, and duodenum as a whole. To date, many studies have focused on the occurrence of anxiety, depression, and nervousness in patients with gastric cancer. Indeed, a previous study found that one-third of patients with gastric cancer had anxiety before admission, while more than half had anxiety during hospitalization [17]. Moreover, FKBP5 polymorphism is associated with anxiety and depression in patients with advanced gastric cancer [18]. It has been shown that depression can lead to oxidative stress, and excessive ROS production impairs the clearance ability of the human body, which may lead to primary gastric cancer. However, the causal relationship between negative emotions and gastric cancer remains unclear.

Through our research, we found no causal relationship between negative emotions and gastric cancer. However, the sample size of patients with gastric cancer in our study was too small, which inevitably led to errors. We need to expand the sample size before evaluation. Some scholars have analyzed the risk factors related to precancerous lesions of gastric cancer in eastern China and found that anxiety and depression, as negative emotions, are related to precancerous lesions of gastric cancer [19]. Gastroduodenal ulcer is a precancerous lesion of gastric cancer, and whether there is a cause-and-effect relationship between negative emotions is another question we hope to address.

The results show that besides nervousness, a cause-and-effect relationship exists between anxiety, depression, and gastroduodenal ulcers. Previous studies on the comorbidity of anxiety have reported that patients with anxiety and depression have an increased risk of ulcers [20]. Some scholars have attempted to explain this through inflammation, the hypothalamus–pituitary–adrenal axis, and other neuroendocrine reactions [21, 22]. However, there may be a two-way relationship between negative emotions and diseases [23]; therefore, the causal relationship between negative emotions and diseases remains unclear. Here, for the first time, we confirm the causal relationship between negative emotions and gastroduodenal ulcers using MR analysis. At present, no previous study has investigated the causal relationship between the nervous system and gastroduodenal ulcers; our results suggest that there is no causal relationship between these two factors. We also studied the relationship between negative emotions and upper gastrointestinal diseases. Previous studies have found that depression and anxiety are often accompanied by gastroesophageal reflux [24]. At the same time, patients with gastroesophageal reflux are more likely to experience anxiety and depression, suggesting a causal relationship between negative emotions and gastroesophageal reflux [25]. First of all, the study included only people of European origin, whether there are genetic differences between different nationalities, countries and regions; secondly, due to the lack of detailed clinical information, subgroup analysis cannot be performed, and thus the specific causal relationship cannot be determined. Animal experiment has shown that the presence of mast cells in the gastric submucosa is associated with pain and psychological diseases. Therefore, it is assumed that activation of these mast cells stimulates vagus nerve activity and leads to changes in psychological behavior by affecting the central nervous system, seemingly providing a theoretical basis for the impact of emotions on gastric disease [26]. However, as a mouse model was used in this study, whether the same mechanism of action is observed in humans requires further study. We also found a causal relationship between depression and esophageal, gastric, and duodenal diseases, while previous studies have shown that duodenal diseases are associated with negative emotions.

## Conclusions

Our findings provide a platform for researchers to explore the relationship between negative emotions and upper gastrointestinal disease. In addition to neuroticism, there is a causal relationship between anxiety, depression and gastroduodenal ulcer. Previous studies on anxiety comorbidity have reported that patients with anxiety and depression have an increased risk of ulcers. Through the analysis of Mendel ’s randomization research method, it is found that negative emotions are positively correlated with the risk of upper gastrointestinal diseases. Future research should clarify the mechanism of the relationship between negative emotions and upper gastrointestinal diseases or explore ways to prevent and treat upper gastrointestinal diseases in patients with negative emotions.

## Supporting information

S1 Table Source of experimental data. (PDF)

S2 Table The data after Mendelian randomization. (CSV)

S3 Table SNPs removed by Phnoscanner. (PDF)

S1 Fig Odds radio plot for Impact of negative emotions on upper gastrointestinal diseases. (TIFF)

S2 Fig Pattern of heterogeneity. (TIFF)

S3 Fig Leave-one-out plot. (TIFF)
