# Supplementary material for: Impact of negative emotions on upper gastrointestinal diseases: A Mendel randomization study
Source: PLoS One. 2024 Jul 12;19(7):e0304121. doi: 10.1371/journal.pone.0304121 (PMC11244763; doi:10.1371/journal.pone.0304121)
Supplement: S1 Table — (PDF) [file pone.0304121.s001.pdf]

Phenotype	GWAS ID
**Anxiety**	ukb-b-18336
**Depression**	ebi-a-GCST005902
**Nervous**	ebi-a-GCST006948
**Gastric cancer**	finn-b-C3_STOMACH
**Gastroduodenal ulcer**	finn-b-K11_GASTRODUOULC
**Diseases of oesophagus, stomach and duodenum**	finn-b-K11_OESSTODUO
