# Supplementary material for: Impact of negative emotions on upper gastrointestinal diseases: A Mendel randomization study
Source: PLoS One. 2024 Jul 12;19(7):e0304121. doi: 10.1371/journal.pone.0304121 (PMC11244763; doi:10.1371/journal.pone.0304121)
Supplement: S3 Table — (PDF) [file pone.0304121.s003.pdf]

rs10035449	26	rs2759663
rs10148293	27	rs2853779
rs10501320	28	rs28820925
rs10747488	29	rs30266
rs10767735	30	rs3132685
rs10960103	31	rs4129585
rs11030107	32	rs4787491
rs117618307	33	rs4919695
rs11877758	34	rs4936275
rs11961509	35	rs510339
rs12417293	36	rs55726687
rs12729445	37	rs62062288
rs1368549	38	rs62212171
rs1375545	39	rs660879
rs17175713	40	rs66511648
rs17884466	41	rs6919397
rs1791684	42	rs7026674
rs1808361	43	rs7030813
rs1890946	44	rs707916
rs1900599	45	rs7227069
rs1986868	46	rs7413892
rs2276882	47	rs7939451
rs2517601	48	rs9267084
rs2568958	49	rs9614090
rs2587505	50	rs9688806
	51	rs9879090
